# Detection of Dysbiosis and Increased Intestinal Permeability in Brazilian Patients with Relapsing–Remitting Multiple Sclerosis

**DOI:** 10.3390/ijerph18094621

**Published:** 2021-04-27

**Authors:** Felipe Papa Pellizoni, Aline Zazeri Leite, Nathália de Campos Rodrigues, Marcelo Jordão Ubaiz, Marina Ignácio Gonzaga, Nauyta Naomi Campos Takaoka, Vânia Sammartino Mariano, Wellington Pine Omori, Daniel Guariz Pinheiro, Euclides Matheucci Junior, Eleni Gomes, de Gislane Lelis Vilela Oliveira

**Affiliations:** 1 Microbiome Study Group, School of Health Sciences Dr. Paulo Prata, Barretos 14785-002, Brazil; fppellizoni@gmail.com (F.P.P.); neuroclinicabarretos@uol.com.br (M.J.U.); marina.ignacio92@hotmail.com (M.I.G.); nauytatakaoka@gmail.com (N.N.C.T.); 2 Microbiology Program, Institute of Biosciences, Humanities and Exact Sciences, São Paulo State University, Sao Jose do Rio Preto 15054-000, Brazil; allline1@hotmail.com (A.Z.L.); eleni.gomes@unesp.br (E.G.); 3 DNA Consult Genetics and Biotechnology, Sao Carlos 13560-340, Brazil; nathdecampos@gmail.com; 4 Barretos Cancer Hospital, Barretos 14784-400, Brazil; vaniasmariano@gmail.com; 5 Department of Technology, School of Agricultural and Veterinarian Sciences, São Paulo State University (UNESP), Jaboticabal 14884-900, Brazil; wpomori@gmail.com (W.P.O.); dgpinheiro@gmail.com (D.G.P.); 6 Biotechnology Department, Sao Carlos Federal University, Sao Carlos 13565-905, Brazil; matheucci@dnaconsult.com.br; 7 Food Engineering and Technology Department, São Paulo State University (UNESP), Sao Jose do Rio Preto 15054-000, Brazil

**Keywords:** autoimmunity, multiple sclerosis, gut microbiota, dysbiosis, inflammation, cytokines, intestinal permeability, disease modifying drugs

## Abstract

Dysbiosis, associated with barrier disruption and altered gut–brain communications, has been associated with multiple sclerosis (MS). In this study, we evaluated the gut microbiota in relapsing–remitting patients (RRMS) receiving disease-modifying therapies (DMTs) and correlated these data with diet, cytokines levels, and zonulin concentrations. Stool samples were used for 16S sequencing and real-time PCR. Serum was used for cytokine determination by flow cytometry, and zonulin quantification by ELISA. Pearson’s chi-square, Mann–Whitney, and Spearman’s correlation were used for statistical analyses. We detected differences in dietary habits, as well as in the gut microbiota in RRMS patients, with predominance of *Akkermansia muciniphila* and *Bacteroides vulgatus* and decreased *Bifidobacterium*. Interleukin-6 concentrations were decreased in treated patients, and we detected an increased intestinal permeability in RRMS patients when compared with controls. We conclude that diet plays an important role in the composition of the gut microbiota, and intestinal dysbiosis, detected in RRMS patients could be involved in increased intestinal permeability and affect the clinical response to DTMs. The future goal is to predict therapeutic responses based on individual microbiome analyses (personalized medicine) and propose dietary interventions and the use of probiotics or other microbiota modulators as adjuvant therapy to enhance the therapeutic efficacy of DMTs.

## 1. Introduction

Multiple sclerosis (MS) is a chronic inflammatory, neurodegenerative disease, mediated by autoimmune reactions against myelin proteins and gangliosides in white and grey matter of the brain and spinal cord, promoting physical disability, cognitive impairment, and decreased quality of life in young adults, aged between 20 and 40 years [1,2]. The incidence of MS is increasing worldwide and estimated to range from 5 to 300 per 100,000 individuals, affecting females three times more and having a significant socioeconomic impact, with financial burden to patients and to developed and developing economies [2,3].

The MS onset is clinically characterized as relapsing–remitting (RRMS), diagnosed in 85 to 90% of patients [1,4]. The relapses are due to blood–brain barrier breakdown and infiltration of T and B cells and myeloid cells into the central nervous system (CNS) parenchyma, which induces acute inflammation, detected as gadolinium-positive lesions in magnetic resonance imaging (MRI) [3]. Permanent neurological lesions and clinical disability evolve to a secondary progressive form, and few patients present a primary progressive course from disease onset [1]. Complex genetic–environmental interactions are hypothesized to be involved in MS development, including human leukocyte antigen (HLA) genes, Epstein–Barr virus infections, tobacco exposure, obesity, vitamin D deficiency, and alterations of the gut microbiota [1,5,6].

In homeostatic or eubiosis conditions, the gut microbiota is dominated by microorganisms that contribute to food digestion and fermentation, nutrient absorption, vitamin synthesis, epithelial cell maturation, gut barrier integrity, development and education of the immune system, protection against pathogens and inflammation, and regulation of host metabolism and CNS physiology [7,8,9,10]. Recently, it has become evident that the gut microbiota can affect neurologic processes through bidirectional communications, involving the enteric nervous system, the endocrine/immune systems, the gut microbiota, and their metabolites [10,11,12,13]. Neurotransmitters and short-chain fatty acids (SCFAs), derived from microbiota fermentation, can shape immune responses and impact behavior, memory, and neurodegenerative diseases [10,12,14,15]. Thus, alterations in function and diversity of the gut microbiota, known as dysbiosis, are associated with a dysregulation in these gut–brain connections, increased gut and blood–brain barrier permeability and neuroinflammation and can contribute to the development of inflammatory autoimmune diseases, including MS [16,17,18,19].

In MS animal models, when experimental autoimmune encephalomyelitis (EAE) was induced in germ-free mice, a decrease in inflammatory interferon-gamma (IFN-γ) and interleukin-(IL)-17A levels in the CNS was detected, as well as an increase in regulatory T cells (Treg) in the gut mucosa [20]. On the other hand, the colonization of EAE mice with segmented filamentous bacteria induced Th17 differentiation in the lamina propria and migration to the CNS, increasing neuroinflammation and disease severity [20,21]. The disease score ameliorated when germ-free EAE mice were colonized by *Bacteroides fragilis* containing polysaccharide A*,* which induces IL-10-secreting Treg cells and suppress the T-helper (Th)-17 subpopulation [22,23]. Moreover, when fecal samples from MS patients were transferred to germ-free mice, genetically susceptible to EAE, the mice developed the disease and significantly produced less IL-10 than mice colonized with feces from healthy subjects [24]. These data suggest that the gut microbiota is linked to disease severity and immune response during MS development [10].

In humans, the gut microbiota from untreated RRMS patients, from different populations (China, Japan, Germany, USA), differs from that of healthy controls, and patients with active disease present decreased microbiota diversity. Intestinal dysbiosis in MS was predominantly characterized by decreased Firmicutes, Clostridia clusters XIVa and IV, *Faecalibacterium*, *Butyricimonas*, *Prevotella*, and *Lactobacillus* species, and increased abundance of *Pseudomonas*, *Mycoplasma*, *Haemophilus*, *Streptococcus*,*Akkermansia muciniphila*, and *Methanobrevibacter smithii* [24,25,26,27,28,29,30,31,32,33]. In addition, MS patients with increased peripheral Th17 lymphocytes and higher disease activity presented an increased Firmicutes/Bacteroidetes ratio, *Streptococcus* amounts, and decreased relative abundance of *Prevotella* species [34]. Interestingly, the taxonomic composition during remission showed richness and evenness similar to those of healthy individuals, and even the frequency of relapses seemed to be influenced by the intestinal microbiota [29,35].

There are few studies evaluating the effect of disease-modifying therapies (DMTs), used to treat MS patients on intestinal microbiota composition. Some studies suggest that these therapies are capable of reversing dysbiosis and restore a “healthy” gut microbiota, similar to that of control subjects [19]. Patients on IFN-β or glatiramer acetate treatment showed increased abundance of *Prevotella, Sutterella*, and *Prevotella copri* and decreased *Sarcina* species and gut microbiota richness [29,36,37]. Besides that, evidence from animal models and human studies demonstrated that gut microbes and their metabolites can influence drug bioavailability, pharmacokinetics, clinical response, as well as adverse events, supporting the importance of studies on the interaction of the gut microbiota with DMTs [38,39]. The future goal is to predict therapeutic responses based on microbiome analyses and propose diet interventions and the use of probiotics or other microbiota modulators as adjuvant therapy to enhance the therapeutic efficacy of DMTs [40,41].

On the basis of this background and the fact that there are no studies evaluating the gut microbiota in Brazilian MS patients, the aim of the present study was to evaluate the gut microbiota in RRMS patients receiving DMTs and correlate these data with dietary habits, clinical parameters, cytokines, and zonulin concentrations.

## 2. Materials and Methods

### 2.1. Selection of Relapsing–Remitting MS Patients and Controls 

Relapsing–remitting multiple sclerosis (RRMS) patients, diagnosed according to the Poser and colleagues criteria [42], were selected by the Neurologist from the School of Health Sciences Dr. Paulo Prata, Barretos, Sao Paulo, Brazil. The Ethics Committee on Human Research from the Barretos Educational Foundation approved the present study (Process number 1522.762/2016), and all subjects signed the informed consent in accordance with the Declaration of Helsinki.

A total of 18 RRMS patients, 16 females and 2 males (mean age − standard deviation (SD) = 46.06 − 11.83 years), were included in this study. Eighteen control subjects, age-and-sex-matched, were included as a control group (mean age − SD = 45.50 − 11.03 years). After the consent, all of subjects answered a food frequency questionnaire (FFQ) that was designed by specialized nutritionists. The FFQ included questions concerning dietary habits, such as consumption of vegetables, fruits, carbohydrates, animal-derived proteins, saturated and trans fats, dairy products, and canned products. The options for frequency of consumption in the FFQ was classified as (1) Never consumes; (2) Less than once a month; (3) One to three times a month; (4) Once or twice a week; (5) Three to five times a week; (6) Six to seven times a week. Data were expressed in percentages based on the responses of patients and controls. Thereafter, peripheral blood (8 mL) was collected, and stool samples were requested and delivered within five days.

At enrollment, exclusion criteria for patients and controls included use of antibiotics and laxatives and vaccination in the last 60 days. Chronic diarrhea and gastrointestinal surgeries, such as bariatric, cholecystectomy, and appendectomy, were also considered as exclusion criteria for both groups.

Clinical data from MS patients, such as body mass index (BMI), disease duration, Expanded Disability Status Score (EDSS), presence/absence of gadolinium (Gd)-enhanced brain magnetic resonance imaging (MRI) lesions, and disease-modifying therapies (DMTs) were recorded. The mean body mass index of the MS patients was 26. Three patients reported having systemic arterial hypertension, and two patients reported taking vitamin D. All other patients included in this study reported no other comorbidity. Demographic characteristics and clinical data from RRMS patients are summarized in Table 1.

### 2.2. Bacterial DNA Extraction, Real-Time PCR, and 16S Sequencing

DNA was extracted from 200 mg of stool samples by using QIAamp DNA Stool Mini Kit (QIAGEN, Hilden, Germany), according to the manufacturer’s instructions. DNA was quantified by Nanodrop and adjusted to 5 ng/mL. Primers were specific for *Bacteroides*, *Bifidobacterium*, *Lactobacillus*, *Prevotella*, and *Roseburia* species [43]. Reactions were performed by using Power SYBR Green PCR Master Mix (Applied Biosystems, Life Technologies, Carlsbad, CA, USA), 2 uM of forward/reverse primers, and 5 ng of DNA. For relative quantification, DNA copy numbers from target primers were normalized for the copy numbers of universal primer. The relative abundance was calculated by using the cycle-threshold (Ct) values and was expressed by the relative expression units method (REU) [44], per 200 mg of stool.

For bacterial 16S sequencing, DNA was quantified by Quantus fluorometer and adjusted to 5 ng/mL using Tris buffer (10 mM, pH 8.5). V3 and V4 regions of the bacterial 16S [45] were amplified by using bacterial DNA, V3/V4 primers, and the 2X KAPA HiFi HotStart Ready Mix (Kapa Biosystems, MA, USA). PCR purification was performed using AMPure XP Beads Kit (BD Biosciences, San Jose, CA, USA). DNA libraries were constructed according to the Illumina protocols, and sequencing was conducted by an Illumina MiSeq platform system.

### 2.3. Cytokine Determination by Cytometric Bead Array

After peripheral blood collection (8 mL) in gel tubes with clot activator, samples were incubated for 50 min and then centrifuged at 1372 g for 5 min, 25 °C. Isolated serum samples were stored until cytokine determination. Cytokine detection was performed by using a cytometric bead array (Human Th1/Th2/Th17 Cytokine Kit, BD Biosciences, Franklin Lakes, NJ, USA). Serum levels of IL-2, IL-4, IL-6, IL-10, IL-17A, tumor necrosis factor (TNF), and IFN-γ were determined by flow cytometer FACSCanto™ II (BD Biosciences). Analyses were performed by BDFCAP array™ software, and data were expressed in pg/mL.

### 2.4. Zonulin Serum Quantification by Sandwich ELISA

Serum samples were isolated from peripheral blood collected in gel tubes with clot activator. After collection, samples were incubated for 50 min, centrifuged at 1372 g for 5 min, and stored until zonulin determination. A human Zonulin ELISA Kit (Elabscience, MD, USA) was used to quantify zonulin concentrations. Plates were pre-coated with antibodies to human zonulin, and serum samples and standards were incubated for 1 h, 37 °C. Then, incubation with biotinylated detection antibodies and avidin–horseradish peroxidase conjugate was performed for 30 min. Three washing steps followed to remove unbound and free molecules. The substrate solution was added to each well and incubated for 15 min. The enzyme–substrate reaction was blocked by a stop solution, and the color turned yellow. The optical density was measured in a spectrophotometer at 450 nm. A standard curve was constructed, and zonulin concentrations were calculated by converting the obtained optical density in ng/mL.

### 2.5. Statistical Analyses

Data extracted from the FFQ were analyzed by Pearson’s chi-square. Comparisons between relative expression units and cytokines’ concentrations in MS patients and controls were performed by a nonparametric Mann–Whitney U test. Zonulin concentrations were analyzed by unpaired *t* test with Welch’s correction, since the data presented < Gaussian distribution. Correlations among the read percentages of the gut microbiota, cytokines, and zonulin concentrations were performed by Spearman’s correlation.

We performed analyses of variance and obtained rarefaction curves and diversity indexes by using annotated operational taxonomic units (OTUs). Alpha diversity summarizes the microbial diversity within each sample, and beta diversity measures differences between samples. Sequencing analysis of bacterial 16S was conducted as described in a previous study [46]. *p* values less than 0.05 were considered statistically significant.

## 3. Results

### 3.1. Dietary Habits and Correlations with the Gut Microbiota in RRMS Patients

Since diet plays a significant role in gut microbiota composition, we used an FFQ in order to detect differences in dietary habits between RRMS patients and healthy controls. The interviewees reported daily consumption of vegetables (patients (Pt) = 77.8%; controls (Ct) = 61.1%), fruits (Pt = 44.4%; Ct = 27.8%), carbohydrates (Pt = 61.1%; Ct = 61.1%), animal-derived proteins (Pt = 50.0%; Ct = 27.8%), saturated/trans fats (Pt = 5.5%; Ct = 16.7%), dairy products (Pt = 55.6%; Ct = 72.2%), and canned products (Pt = 0.0%; Ct = 5.5%). We observed significant differences (*p* < 0.05) among intake of vegetables, fruits, carbohydrates, animal-derived proteins, and dairy products when we compared patients and controls. Table 2 summarizes the data obtained from the FFQ, with the frequencies of food consumption per patient and controls and the *p* values.

To find correlations between dietary habits and gut microbiota composition in RRMS patients, we used the consumption frequencies and the reads percentages detected in stool samples from RRMS patients. We detected significant moderate/strong correlation between vegetables consumption by patients and relative abundance of *Roseburia* (*p* = 0.010; *r* = −0.60). We also found negative correlations between animal-derived protein intake and relative abundance of Verrucomicrobiae/Verrucomicrobiales (*p* = 0.041; *r* = −0.50) and *Bacteroides vulgatus* (*p* = 0.014; *r* = −0.58).

### 3.2. Detection of Intestinal Dysbiosis and Prevalence of Gram-Negative Bacteria in RRMS Patients

**Figure 1:**
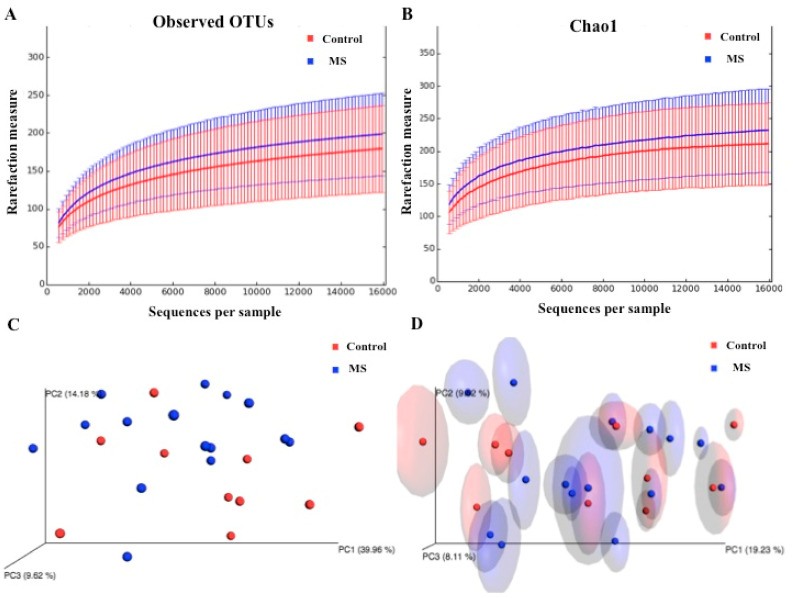
Alpha and beta diversity in the gut microbiota of RRMS patients receiving DMTs and that of healthy controls. Rarefaction curves are a representation of species richness for a given number of individual samples: (**A**) Observed and (**B**) Chao 1-estimated OTUs. Principal component analysis (PcoA) is a transformation of weighted or unweighted Unifrac distance, a pair-wise distance between samples based on the calculation of the shared branches of the phylogenetic tree of the representative rRNA genes from OTUs present in at least one sample: (**C**) PcoA plot with weighted and (**D**) unweighted UniFrac metric with Bonferroni’s correction.

For the purpose to detect intestinal dysbiosis in RRMS patients receiving DMTs, we sequenced the V3/V4 regions from bacterial 16S and determined the alpha and beta diversities by using the annotated operational taxonomic units (OTUs). According to the rarefaction curves, we observed no significant differences (*p* = 0.38) in richness and evenness between samples obtained from RRMS patients and controls (Figure 1A,B). However, when we used the unweighted UniFrac metric with Bonferroni correction, we detected a significant difference (*p* = 0.01) between microbial communities found in RRMS patients and controls (Figure 1D). Figure 1C shows the PcoA plot regarding the weighted UniFrac metric with Bonferroni correction.

**Figure 2:**
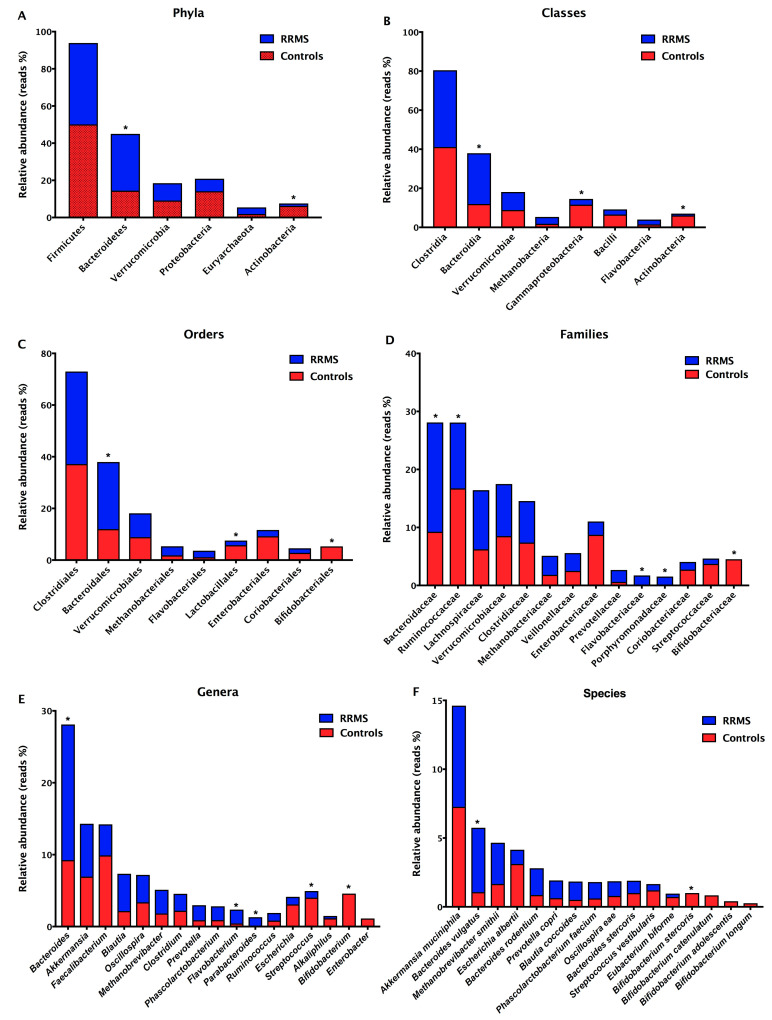
Relative abundances of bacterial taxa in stool samples from RRMS patients and controls. Predominant phyla (**A**), classes (**B**), orders (**C**), families (**D**), genera (**E**), and species (**F**). Bars represent the reads percentages found in metagenomics analyses. * *p* < 0.05.

To compare the microbiota composition in treated RRMS patients and controls, we sequenced the bacterial 16S in stool samples and analyzed specific bacterial groups by real-time PCR. The prevalent phyla in RRMS patients were Firmicutes (patient reads (Pr) = 43.78%; control reads (Cr) = 50.12%) and Bacteroidetes (Pr = 30.52%; Cr = 14.47%), and the prevalent classes were Clostridia (Pr = 39.29%; Cr = 41.15%) e Bacteroidia (Pr = 25.96%; Cr = 11.99%) (Figure 2A,B). The prevalent orders were Clostridiales (Pr = 35.80%; Cr = 37.16%) and Bacteroidales (Pr = 25.96%; Cr = 11.99%), and the prevalent families were Bacteroidaceae (Pr = 18.86%; Cr = 9.25%), Ruminococcaceae (Pr = 11.35%; Cr = 16.74%), and Lachnospiraceae (Pr = 10.19%; Cr = 6.24%) (Figure 2C,D). The prevalent genera in RRMS patients were *Bacteroides* (Pr = 18.86%; Cr = 9.25%), *Akkermansia* (Pr = 7.35%; Cr = 6.95%), *Blautia* (Pr = 5.18%; Cr = 2.16%), and *Faecalibacterium* (Pr = 4.31%; Cr = 9.91%). The prevalent species in stool samples from RRMS patients were *Akkermansia muciniphila* (Pr = 7.35%; Cr=7.27%), *Bacteroides vulgatus* (Pr = 4.68%; Cr = 1.07%), *Methanobrevibacter smithii* (Pr = 2.99%; Cr = 10.01%)*, Bacteroides rodentium* (Pr = 1.95%; Cr = 3.43%), *Blautia coccoides* (Pr = 1.33%; Cr = 2.05%), and *Prevotella copri* (Pr = 1.28%; Cr = 1.09%) (Figure 2E,F). Additionally, we found significant differences (*p* < 0.05) in the relative abundances of Bacteroidetes and Actinobacteria phyla, Bacteroidia, Gammaproteobacteria and Actinobacteriia classes, Bacteroidales, Lactobacillales, and Bifidobacteriales orders, Bacteroidaceae, Ruminococcaceae, Flavobacteriaceae, Porphyromonadaceae, and Bifidobacteriaceae families, *Bacteroides*, *Flavobacterium*, *Parabacteroides*, *Streptococcus, Bifidobacterium* genera, *Bacteroides vulgatus* and *Bifidobacterium stercoris* between samples derived from patients and controls (Figure 2). Interestingly, the *Parabacteroides* genus (Pr = 1.31%; Cr = 0%) was detected only in stool samples from RRMS patients, and the *Bifidobacterium* (Pr = 0%; Cr = 4.59%) and *Enterobacter* (Pr = 0%; Cr = 1.12%) genera were found exclusively in stool samples from controls (Figure 2E).

**Figure 3:**
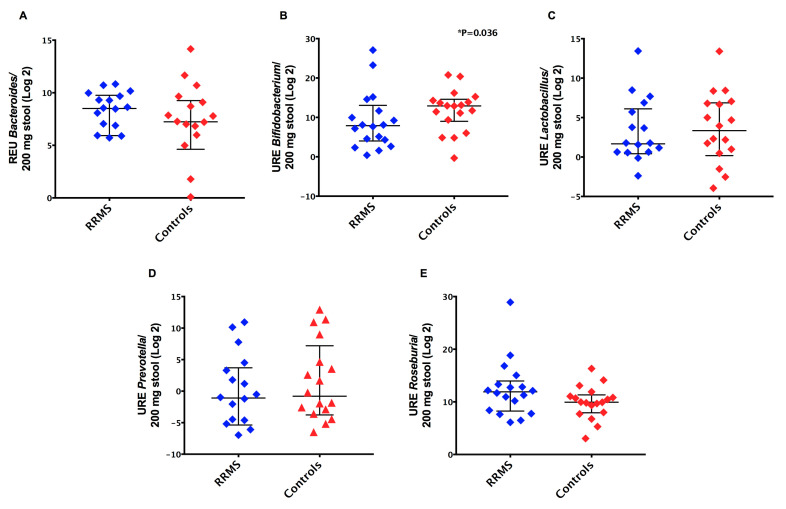
Relative abundance of bacterial community in stool samples from RRMS patients and controls. (**A**) *Bacteroides* species, (**B**) *Bifidobacterium* species, (**C**) *Lactobacillus* species, (**D**) *Prevotella* species, and (**E**) *Roseburia* species. Bars represent the median with interquartile range of relative expression units (REU) per 200 mg of stool.

Regarding the characterization of the gut microbiota by real-time PCR, we observed similar relative expression units (*p* > 0.05) of *Bacteroides, Lactobacillus, Prevotella,* and *Roseburia* species when we compared patients’ and controls’ samples (Figure 3). In contrast, we found a significant decrease (*p* = 0.036) in relative expression units of *Bifidobacterium* species detected in stool samples derived from RRMS patients (median = 239.7) compared to controls (median = 7791) (Figure 3B). Moreover, when we classified MS patients based on different DMTs, there were no significant differences (*p* > 0.05) in relative expression units of *Bacteroides*, *Bifidobacterium*, *Clostridium coccoides*, *Clostridium coccoides-Eubacterium rectale*, *Clostridium leptum*, *Lactobacillus*, *Prevotella*, and *Roseburia* in stool samples from MS patients.

### 3.3. Detection of Decreased Pro-Inflammatory IL-6 Cytokine in MS Patients

**Figure 4:**
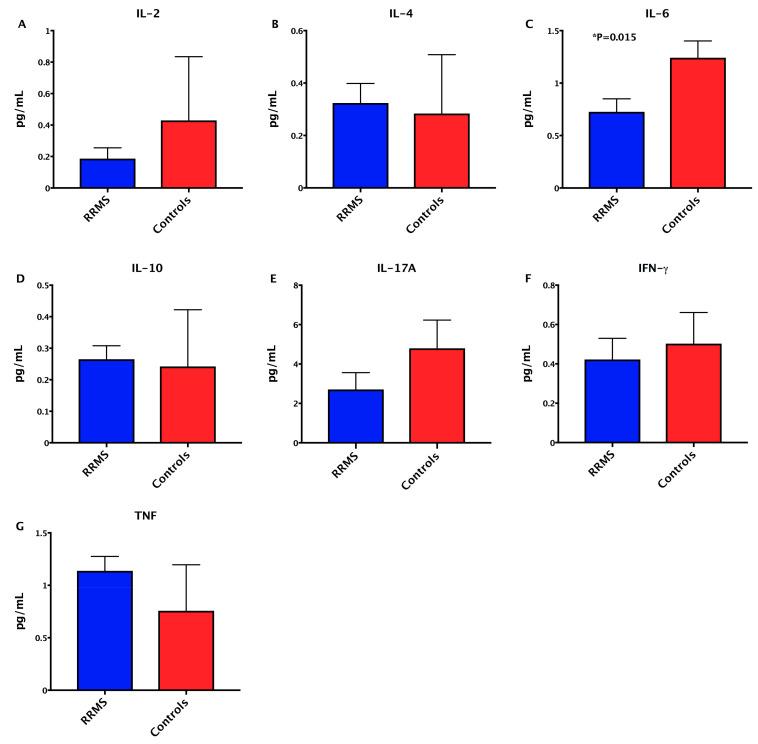
Cytokine profile in treated RRMS patients and control subjects. Serum concentrations of (**A**) IL-2, (**B**) IL-4, (**C**) IL-6, (**D**) IL-10, (**E**) IL-17A, (**F**) IFN-gamma, and (**G**) TNF. Statistical analyses were performed by the Mann–Whitney test. Significance was set at *p* < 0.05.

**Figure 5:**
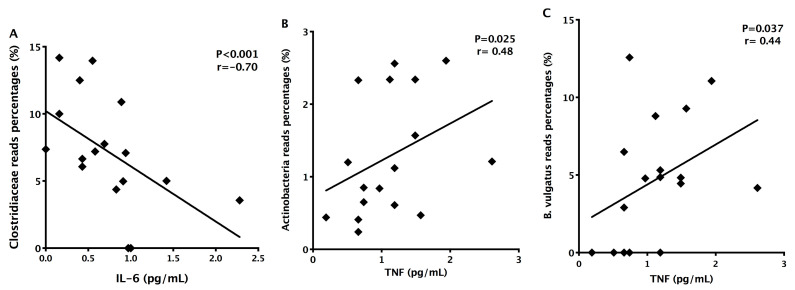
Correlations among relative abundances of bacterial taxa and serum concentrations of inflammatory cytokines. (**A**) Negative correlation between relative abundance of Clostridiaceae and IL-6 concentrations in RRMS patients; (**B**) Positive correlation between relative abundance of Actinobacteria and TNF concentrations; (**C**) Positive correlation between *Bacteroides vulgatus* and TNF concentrations. Statistical analyses were performed by Spearman’s test. Significance was set at *p* < 0.05.

To determine the serum concentrations of anti- and pro-inflammatory cytokines in RRMS patients, we quantified IL-2, IL-4, IL-6, IL-10, IL-17A, IFN-gamma, and TNF by cytometric bead array. There were no significant differences (*p* < 0.05) in the concentrations of IL-2, IL-4, IL-10, IL-17A, TNF in patients’ serum (mean ± standard error IL-2: 0.1867 ± 0.0687 pg/mL; IL-4: 0.3239 ± 0.0743 pg/mL; IL-10: 0.265 ± 0.0429 pg/mL; IL-17A: 2.708 ± 0.8544 pg/mL; TNF: 1.138 ± 0.1372 pg/mL; IFN-gamma: 0.4222 ± 0.1076 pg/mL) when compared with control group (IL-2: 0.4294 ± 0.4051 pg/mL; 233IL-4: 0.2839 ± 0.2244 pg/mL; IL-10: 0.2422 ± 0.18 pg/mL; IL-17A: 4.796 ± 1.43 pg/mL; TNF: 0.7572 ± 0.4383 pg/mL; IFN-gamma: 0.5028 ± 0.158 pg/mL) (Figure 4A–G). IL-6 serum concentrations were decreased (*p* = 0.003) in RRMS patients (0.7261 ± 0.1244 pg/mL) when compared with controls (1.242 ± 0.1601 pg/mL) (Figure 4C). In addition, IL-6 concentrations inversely correlated with Clostridiaceae family members (*p* = 0.001; *r* = −0.70), and TNF levels correlated with Actinobacteria (*p* = 0.025; *r* = 0.48) and *Bacteroides vulgatus* (*p* = 0.001; *r* = −0.70) (Figure 5A–C).

### 3.4. Detection of Increased Intestinal Permeability in RRMS Patients

**Figure 6:**
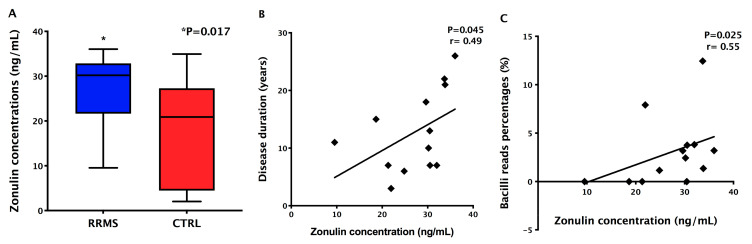
Zonulin concentrations and correlations with clinical data and gut microbiota. (**A**) Serum zonulin concentrations in RRMS patients and controls (CTRL); (**B**) Positive correlation between zonulin concentrations and disease duration; (**C**) Positive correlation between zonulin concentrations and relative abundance of Bacilli class members.

In order to find whether RRMS patients presented increased intestinal permeability, since alterations in the gut microbiota were detected, we evaluated the serum concentrations of zonulin. Zonulin levels were significantly increased (*p* = 0.017) in MS patients’ samples (mean ± standard error: 27.13 ± 2.08 ng/mL) when compared with controls’ (mean ± standard error: 19.01 ± 2.98 pg/mL) (Figure 6A). Besides that, zonulin concentrations positively correlated with disease duration (*p* = 0.025; *r* = 0.55; Figure 6B) and with the relative abundance of Bacilli class members (*p* = 0.045; *r* = 0.49; Figure 6C) in MS patients.

## 4. Discussion

The dietary habits in industrialized societies have considerable changed in the last years, and concomitant to this changes, the frequency of autoimmune diseases has increased [47]. Western diets include low fiber and high fat consumption, which alters the gut microbiota diversity and function, affecting the mucosal immune system and influencing the development of autoimmune diseases [48]. Berer and colleagues (2018) demonstrated that the supplementation of non-fermentable fiber to transgenic mice of the spontaneous EAE model (opticospinal encephalomyelitis mice) impacted gut microbiota and metabolic profile, increased long-chain fatty acids production, induced polarization to Th2 immune responses, and prevented autoimmune diseases [49]. Furthermore, exercise practice and low-calorie diets based on the consumption of vegetables, fruits, fish, prebiotics, and probiotics induced a decrease in inflammatory mediators and reestablished eubiosis by acting via nuclear receptors [50]. Additionally, Wu and colleagues (2011) showed the influence of diet on the gut microbiota and the prevalence of *Bacteroides* species when animal proteins and saturated fats were consumed, while the presence of *Prevotella* species was associated with carbohydrates and simple sugar intake [51]. In our study, we detected significant differences in the consumption of vegetables, fruits, carbohydrates, animal-derived proteins, and dairy products between patients and controls and, in contrast to Wu et al., we detected an inverse correlation between increased animal-derived protein intake by patients (50% vs. 27.8% in controls) and relative abundance of *Bacteroides vulgatus*. There are no studies evaluating the intestinal microbiota of the Brazilian population as a whole, and it should be noted that the human intestinal microbiota is considered to be variable between individuals and presents geographic variation [52].

Several clinical trials are underway to test the effects of dietary interventions on inflammatory diseases, such as MS (NCT03539094, NCT02580435, NCT04574024, NCT04042415, NCT03451955). So far, protective effects have been proposed for a Mediterranean diet enriched in fibers, vegetables, polyunsaturated fatty acids, and low levels of proteins [51,53]. On the other hand, the consumption of large amounts of milk and derivatives, meat, or animal fats correlates with an increasing prevalence of MS [54]. We detected differences in dairy products consumption between patients and controls, and inverse correlations with Bacteroidetes members, carbohydrate-degrading, Gram negatives bacteria, including *Bacteroides uniformis* [55]. In MS patients, it has been suggested that dysbiosis caused by an inadequate diet may indirectly influence Tregs/Th17 cell balance in the gut mucosa and activate inflammatory pathways, contributing to intestinal and systemic inflammation and MS pathogenesis [56]. Although we detected differences in diet and alterations in the gut microbiota, the levels of inflammatory IL-17 and IFN*-γ* cytokines, which are involved in MS pathogenesis [57], were similar in patients and controls. However, we detected a significant decrease in IL-6 levels, which are probably associated with DMTs, which impacts the immune response in relapsing–remitting patients [58].

The gut microbiota and the CNS are connected in a bidirectional manner, including neural, endocrine, and immunological interactions [59]. Commensal microbes can interfere with the secretion of neurotransmitters by intestinal cells, stimulate the vagus nerve thus affecting the brain and behavior, produce neuroactive molecules, and modulate mucosal immune cells and systemic populations that can cross the blood–brain barrier (BBB) into the CNS [60]. In turn, the CNS modulates the microbiota by adrenergic signaling and impacts intestinal motility and neurotransmitters actions in immunological cells that shape the gut microbiota composition [60]. Interestingly, a small fraction of metabolites generated by the gut microbiota in response to diet can reach the systemic circulation, cross the blood BBB through vascular epithelial receptors, and modulate CNS inflammation [10,61,62,63]. Besides that, these metabolites can indirectly act through SCFA receptors in MS patients and through aryl hydrocarbon receptors that influence microglia activation and gene transcription in astrocytes [53,63,64]. In animal models, previous studies showed that germ-free mice with a breakdown of tight-junctions at the BBB had defective permeability, restored when these mice were colonized with conventional microbiota [65]. Therefore, a disbiotic microbiota secretes metabolites that enter the blood stream and impact the development of local and systemic diseases [49]. Moreover, these microbes may influence therapeutic responses by activating or inhibiting exogenous molecules [60].

In the present study, we detected intestinal dysbiosis in RRMS patients receiving DMTs, and our results present some similarities with previous studies in non-treated patients [24,25,26,27,28,29,30,31,32,33]. Some of these similarities include decreased *Lactobacillus* spp. (Lactobacillales) and predominance of *Akkermansia muciniphila* and *Methanobrevibacter smithii,* chemilitotrophic specie. *Methanobrevibacter* is involved in inflammatory conditions by recruiting macrophages and activating dendritic cells [66]. *Akkermansia* have immunoregulatory effects by converting mucin into SCFAs [54]; however, they play a role in the degradation of the mucus layer and can promote intestinal inflammation [56]. In addition, we detected a reduced relative abundance of *Bifidobacterium* spp. and Ruminocaceae members including *Faecalibacterium* spp. and *Ruminococcus* spp. *Bifidobacterium* represents one of the first colonizers of the human gut and exerts health-promoting effects [67]. *Faecalibacterium* spp. are butyrate-producing bacteria in the human colon, a bioindicator of human health, and are reduced in inflammatory conditions [68]. *Ruminococcus* spp. re part of the healthy gut microbiota in humans, and some mucus-degrading species are increased in inflammatory diseases [69].

There are few studies evaluating the effect of DMTs on gut microbiota composition, and previous works suggest that these therapies are able to reestablish the gut ecosystem towards a eubiosis condition [19]. Patients on IFN-β or glatiramer acetate treatment showed increased abundance of *Prevotella, Sutterella*, and *Prevotella copri* and decreased *Sarcina* species [29,36,37]. In our MS patients, we also observed an increase in *Prevotella* spp. (Bacteroidales) in treated RRMS patients. The *Prevotella* genus is associated with a high-fiber diet and has regulatory roles via butyrate generation [28]. Butyrate has anti-inflammatory effects, induces Tregs in the gut mucosa, and maintains the epithelial barrier [70]. It is important to note that metabolites produced by the gut microbiota are capable of influencing drug bioavailability, pharmacokinetics, and clinical response, which supports the importance of studies on the interaction of the gut microbiota with DMTs [38,39]. In our work, the treated RRMS patients had a different microbiota profile when compared with healthy controls, suggesting that the disbiotic microbiota could interfere with the therapeutic response and with intestinal permeability, which was significantly increased in our patients.

In addition to changes in the gut microbiota, recent studies have associated small intestine rupture with the development of MS, and, based on this, Rahman and colleagues hypothesized that a leaky gut is mechanistically linked to BBB disruption through receptors for zonulin [71]. One of the predictors of intestinal permeability in humans is the serum zonulin level. Zonulin is a physiological modulator of tight junctions involved in the traffic of macromolecules and in the maintenance of epithelial barrier integrity and immune tolerance in the gut mucosa. [72]. A leaky gut in mice induces inflammatory cytokines release that promote an increased permeability, establishing a vicious circle favoring the entry of antigens derived from diet and gut microbes, inducing a tolerance breakdown and the activation of immune cells in the gastrointestinal mucosa [73,74]. The activated immune cells can remain in the gut or migrate to distant organs, including the brain [73,74,75].

Intestinal dysbiosis can activate the zonulin pathway and stimulate cytokines release allowing the leakage of luminal contents through the epithelial barrier [73]. A study from Camara-Lemarroy and colleagues detected an increase in serum zonulin concentrations in RRMS patients, which positively correlated with BBB disruption, confirmed by positive gadolinium images in MRI [76]. In the present study, we detected a significant increase in serum zonulin concentrations in treated RRMS patients, suggesting that increased gut permeability could be a consequence of the intestinal dysbiosis detected in treated RRMS patients.

## 5. Conclusions

We conclude that diet plays an important role in the composition of the intestinal microbiota in MS patients and controls. In addition, intestinal dysbiosis, detected in RRMS patients receiving DMTs, could be involved in increased intestinal permeability and affect clinical response, future relapses, and disease progression in MS patients. Additional studies in patients with different forms of MS, using DMTs, in different populations are needed, and the future goal is to predict therapeutic responses based on individual microbiome analyses (personalized medicine) and propose dietary interventions and the use of probiotics or other microbiota modulators as adjuvant therapy to enhance the therapeutic efficacy of DMTs. 

## Figures and Tables

**Table 1:** Demographic and clinical data of the relapsing–remitting multiple sclerosis patients.

Patients	Gender/ Age	BMI	Ethnicity	Disease Duration	EDSS	MRI	DMT
**MS01**	F/59	23.11	Caucasian	21 years	5.0	Gd-	IFN-*β*-1b
**MS02**	F/62	19.65	Asiatic	22 years	ND	ND	IFN-*β*-1b
**MS03**	F/50	23.33	Afrodescendent	26 years	ND	ND	AZA
**MS04**	F/26	24.44	Caucasian	3.2 years	4.5	Gd+	GA
**MS05**	F/69	23.42	Caucasian	7 years	3.0	Gd-	GA
**MS06**	F/45	34.41	Caucasian	9 years	3.0	Gd+	TER
**MS07**	F/37	26.67	Caucasian	7 years	4.0	Gd-	IFN-*β*-1b
**MS08**	F/33	34.42	Caucasian	10 years	3.0	ND	GA
**MS09**	F/30	22.98	Caucasian	6 years	3.0	Gd+	FTY720
**MS10**	F/57	25.39	Caucasian	15 years	ND	ND	FTY720
**MS11**	M/44	28.40	Caucasian	18 years	4.5	Gd-	IFN-*β*-1a
**MS12**	F/37	23.05	Caucasian	13 years	ND	ND	GA
**MS13**	F/50	23.22	Caucasian	7 years	3.5	Gd+	IFN-*β*-1a
**MS14**	F/33	28.00	Caucasian	3 years	4.0	Gd+	IFN-*β*-1a
**MS15**	F/47	27.05	Caucasian	7 months	2.5	Gd+	IFN-*β*-1b
**MS16**	F/49	23.82	Caucasian	2 years	4.0	Gd+	NAT
**MS17**	F/56	29.41	Caucasian	12 years	ND	ND	FTY720
**MS18**	M/45	29.66	Caucasian	7 years	3.0	Gd-	IFN-*β*-1b

F: Female; M: Male; BMI: Body Mass Index; EDSS: Expanded Disability Status Score; MRI: Magnetic resonance imaging; Gd+: Presence of gadolinium-enhanced brain lesions; ND: not determined; Gd-: Absence of inflammatory active lesions; DMT: Disease-modifying therapy; IFN-*β*-1b: Interferon-*β*-1b; AZA: Azathioprine; GA: Glatiramer acetate; TER: Teriflunomide; FTY720: Fingolimod; NAT: Natalizumab.

**Table 2:** Description of the dietary habits of multiple sclerosis patients and controls.

Consumption Frequency	*N*	RRMS (%)	*N*	Controls (%)	*p* Value
***Vegetables***					
Once or twice a week	2	11.1%	2	11.1%	*p* < 0.001
Three to five days a week	2	11.1%	5	27.8%	
Six to seven days a week	14	77.8%	11	61.1%	
***Fruits***					
One to three times a month	0	0	4	22.2%	*p* = 0.047
Once or twice a week	0	0	5	27.8%	
Three to five days a week	10	55.6%	4	22.2%	
Six to seven days a week	8	44.4%	5	27.8%	
***Carbohydrates***					
Never consumes	1	5.55%	0	0	*p* < 0.001
Less than once a month	2	11.1%	0	0	
One to three times a month	0	0	1	5.5%	
Once or twice a week	1	5.5%	3	16.7%	
Three to five days a week	3	16.7%	3	16.7%	
Six to seven days a week	11	61.1%	11	61.1%	
***Animal-derived proteins***					
Never consumes	0	0	1	5.5%	*p* < 0.001
One to three times a month	1	5.5%	0	0	
Once or twice a week	6	33.4%	8	44.5%	
Three to five days a week	2	11.1%	4	22.2%	
Six to seven days a week	9	50.0%	5	27.8%	
***Saturated/trans fats***					
Never consumes	6	33.4%	2	11.1%	*p* = 0.444
Less than once a month	2	11.1%	6	33.4%	
One to three times a month	3	16.7%	1	5.5%	
Once or twice a week	4	22.2%	5	27.8%	
Three to five days a week	2	11.1%	1	5.5%	
Six to seven days a week	1	5.5%	3	16.7%	
***Dairy products***					
Never consumes	3	16.7%	1	5.5%	*p* < 0.001
Once or twice a week	1	5.5%	2	11.1%	
Three to five days a week	4	22.2%	2	11.1%	
Six to seven days a week	10	55.6%	13	72.2%	
***Canned products***					
Never consumes	7	38.9%	3	16.7%	*p* = 0.083
Less than once a month	5	27.7%	3	16.7%	
One to three times a month	3	16.7%	4	22.2%	
Once or twice a week	3	16.7%	7	38.9%	
Six to seven days a week	0	0	1	5.5%	

The consumption of dairy products by patients correlated with the presence of the Bacteroidetes phylum (*p* = 0.015; *r* = −0.58), Bacteroidia/Bacteroidales (*p* = 0.011; *r* = −0.60), Bacteroidaceae/*Bacteroides* (*p* = 0.016; *r* = −0.57), *Bacteroides rodentium* (*p* = 0.044; *r* = −0.49), and *Bacteroides uniformis* (*p* = 0.049; *r* = −0.48). Furthermore, we reported a positive correlation between saturated/trans fat consumption and the abundance of Firmicutes (*p* = 0.044; *r* = 0.49), Clostridia (*p* = 0.039; *r* = 0.50), and Clostridiales (*p* = 0.035; *r* = 0.51).

## Abbreviations

MS: multiple sclerosis; RRMS: relapsing-remitting MS; CNS: central nervous system; MRI: magnetic resonance imaging; PPMS: primary progressive MS; SPMS: secondary progressive MS; HLA: human leucocyte antigens; SCFAs, short-chain fatty acids; EAE: experimental autoimmune encephalomyelitis; IFN: interferon; IL: interleukin; Treg: regulatory T cells; Th: T helper; DMTs: disease modifying therapies; SD: standard deviation; FFQ: food frequency questionnaire; F: female; M: male; BMI: body mass index; EDSS: expanded disability status score; Gd+: presence of gadolinium-enhancement brain lesions; ND: not determined; Gd-: Absence of inflammatory active lesions; AZA: Azathioprine; GA: Glatiramer acetate; TER: Teriflunomide; FTY720: Fingolimod; NAT: Natalizumab; REU: relative expression units; Ct: cycle threshold; TNF: tumor necrosis factor; OTUs: operational taxonomic units; PcoA: principal component analysis; BBB: blood-brain barrier.
